# Using Vibration for Secure Pairing With Implantable Medical Devices: Development and Usability Study

**DOI:** 10.2196/57091

**Published:** 2025-08-26

**Authors:** Mo Zhang, Chaofan Wang, Weiwei Jiang, David Oswald, Toby Murray, Eduard Marin, Jing Wei, Mark Ryan, Vassilis Kostakos

**Affiliations:** 1 School of Computer Science, University of Birmingham, Birmingham, United Kingdom; 2 School of Computing and Information Systems, The University of Melbourne, Melbourne Connnect, 700 Swanston Street, Carlton, Melbourne, 3053, Australia, 61 493164461; 3 College of Computer Science and Artificial Intelligence, Wenzhou University, Wenzhou, China; 4 School of Computer Science, Nanjing University of Information Science and Technology, Nanjing, China; 5 Telefonica Research Spain, Barcelona, Spain

**Keywords:** implantable medical device, pairing, vibration, security, usability

## Abstract

**Background:**

Implantable medical devices (IMDs), such as pacemakers, increasingly communicate wirelessly with external devices. To secure this wireless communication channel, a pairing process is needed to bootstrap a secret key between the devices. Previous work has proposed pairing approaches that often adopt a “seamless” design and render the pairing process imperceptible to patients. This lack of user perception can significantly compromise security and pose threats to patients.

**Objective:**

The study aimed to explore the use of highly perceptible vibrations for pairing with IMDs and aim to propose a novel technique that leverages the natural randomness in human motor behavior as a shared source of entropy for pairing, potentially deployable to current IMD products.

**Methods:**

A proof of concept was developed to demonstrate the proposed technique. A wearable prototype was built to simulate an individual acting as an IMD patient (real patients were not involved to avoid potential risks), and signal processing algorithms were devised to use accelerometer readings for facilitating secure pairing with an IMD. The technique was thoroughly evaluated in terms of accuracy, security, and usability through a lab study involving 24 participants.

**Results:**

Our proposed pairing technique achieves high pairing accuracy, with a zero false acceptance rate (indicating low risks from adversaries) and a false rejection rate of only 0.6% (1/192; suggesting that legitimate users will likely experience very few failures). Our approach also offers robust security, which passes the National Institute of Standards and Technology statistical tests (with all *P* values >.01). Moreover, our technique has high usability, evidenced by an average System Usability Scale questionnaire score of 73.6 (surpassing the standard benchmark of 68 for “good usability”) and insights gathered from the interviews. Furthermore, the entire pairing process can be efficiently completed within 5 seconds.

**Conclusions:**

Vibration can be used to realize secure, usable, and deployable pairing in the context of IMDs. Our method also exhibits advantages over previous approaches, for example, lenient requirements on the sensing capabilities of IMDs and the synchronization between the IMD and the external device.

## Introduction

### Background

Implantable medical devices (IMDs), such as pacemakers, implantable cardioverter defibrillators, or insulin pumps are widely deployed and evolving at a rapid pace [1]. Modern IMDs typically rely on a wireless interface to communicate with external devices. For instance, doctors use programmer devices to reprogram the patient’s IMD (eg, to change the patient’s therapy) and gather telemetry data. Such wireless connectivity can bring about much convenience to patients and doctors. However, it also poses new security and privacy threats, such as eavesdropping on sensitive medical data or hijacking life-critical functions. The consequences of such attacks can be severe because they can cause serious injuries or even death. However, these risks have often been overlooked. While no real-world attack against an IMD has been confirmed to date, previous research has demonstrated that many IMDs available on the market today severely lack effective security mechanisms, and that attacks on patients would be practically possible [2-6].

To protect wireless communication links, it is essential for the IMD and external device to undergo a pairing process. This process aims to exchange a cryptographic key between them, which can then be used to secure the wireless channel using standard protocols [7]. However, implementing such a key exchange in a secure manner is challenging because IMDs are resource-constrained with limited memory, computational power, and nonrechargeable and nonreplaceable batteries. Moreover, IMDs do not have physically accessible input or output interfaces, such as a keyboard or a screen once they are implanted. This obstructs traditional pairing methods used in technologies like Bluetooth, where manually typing a 4-digit PIN code on the devices is a standard procedure [8]. Furthermore, network connections with these devices can be ad-hoc. For instance, in an emergency (eg, patients with cardiac implants can experience syncope symptoms and become unconscious [9]), a doctor may quickly have to use a new programmer device to connect to the patient’s IMD. Due to these limitations of IMDs, conventional pairing techniques (such as the ones based on symmetric or public keys [10]) are often not a viable option [5,11].

Previous work has proposed a variety of pairing techniques to overcome this challenge [12]. Rasmussen et al [13] propose an approach where the IMD and external device send ultrasound to each other to verify each other’s legitimacy and exchange a key. Marin et al [5] and Tomlinson et al [14] propose a pairing method by transmitting a low alternating current through the patient’s skin and tissue. Denning et al [15] and Gollakota et al [16] propose to delegate security to a proxy device that the patient can carry around (such as a bracelet). [17-20] propose a pairing process by the IMD and external device synchronously and simultaneously measuring a human physiological signal (such as heartbeats).

Across those previous approaches, a crucial aspect has been systematically overlooked: user perception. We observe that previous work has attempted to follow a “seamless” design approach that makes the IMD pairing as unobtrusive as possible to the patient, rendering the pairing process almost imperceptible at the same time. This can prevent patients from detecting unexpected pairing attempts made by adversaries in proximity, thereby hindering their ability to appropriately respond to such security threats, for example, by seeking assistance or fleeing the scene. Although the “seamless” design principle is common in everyday security systems [21], we question its suitability in the IMD context, where the device is part of the patient, and its security is life-critical.

**Figure 1.**
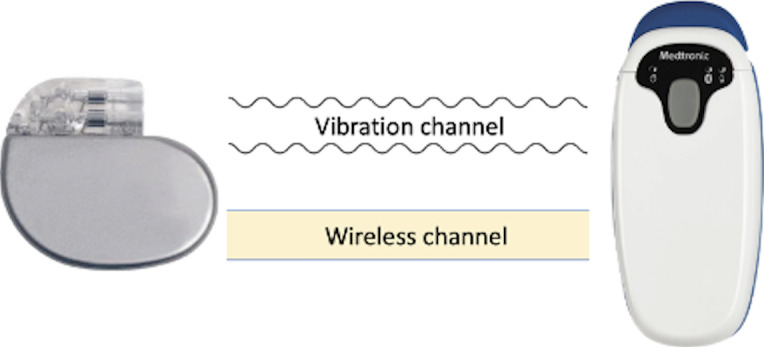
IMD and external device. The vibration channel is used to exchange a key that subsequently secures the wireless channel. IMD: implantable medical device.

To address this issue, a pairing protocol needs to incorporate a perceivable and robust (ie, cannot be hidden or canceled by an adversary) signal. This leads us to consider vibration as an out-of-band (OOB) channel (ie, a communication channel other than a wireless channel) for pairing (Figure 1). Vibrations are highly perceivable and have been widely used in smart consumer devices for notification services [22]. In addition, accelerometers, the primary type of vibration receiver used in previous approaches, are already present in state-of-the-art IMDs for medical purposes [23-25]. Another advantage of using vibration is its limited range of reliable reception. In the IMD context, this implies that if an external device intends to transmit a vibration to an IMD, it must be physically attached to the patient’s skin for a while [26]. If an adversary overpowers the signal with a very strong vibration from a distance, the patient can easily notice this.

### Related Work

#### Vibration-Based Secret Transmission in Ad-Hoc Networks

Previous work has proposed vibration as an OOB channel for transmitting secrets between 2 devices that physically contact each other [26-32]. Table 1 summarizes their application scenarios and hardware setups. Most are designed for wearables and Internet of Things (IoT) devices that are not implanted in the human body. As a common setup, the transmitter (such as a smartphone) is equipped with a vibration motor and the receiver contains a sensor to detect the vibrations, such as an accelerometer [26,27,29-31], gyroscope [32] or microphone [28].

**Table 1.** Setup for previous vibration-based secret transmission. The application context refers to the intended receiver device.

Technique	Application context	Receiver sensor type	Sampling rate (Hz)
Vibrate-to-Unlock [30]	RFID tag	Accelerometer	Not reported
SYNCVIBE [27]	Wearable	Accelerometer	1600
SecureVibe [26]	IMD^a^	Accelerometer	3200
VibroComm [32]	IoT^b^ device	Gyroscope	32000
Ripple [29]	Mobile device	Accelerometer	1600
Ripple II [28]	Mobile device	Microphone	48000
Touch-And-Guard [31]	Wristband device	Accelerometer	250

a IMD: implantable medical device.

b IoT: Internet of Things.

Previous work predominantly directly embeds the secret within the vibration signal itself [26-30,32]: the transmitter encodes the secret into vibration using specific modulation methods (eg, on-off keying [26,27,33]), and the receiver picks up this vibration with a sensor and decodes the secret. Another strategy leverages vibration to “amplify” the secret from humans: Wei et al [31] propose an approach that pairs an IoT device with a wristband device. When the user (who wears the wristband) touches the IoT device, the IoT device emits a vibration that sweeps through a range of frequencies. Contrary to the above methods, the vibration here does not carry the secret and remains consistent across different sessions. Instead, the secret comes from the (to some extent random) resonant properties of the user’s hand-arm area, which can be derived from the devices’ accelerometer readings.

However, we argue that most work (in their current form) is not deployable in existing IMD products because they have stringent requirements on the receiver sensor. Microphones do not exist in IMDs, while inertial sensors (ie, accelerometer and gyroscope) often require sampling rates in several thousands of Hz or higher. Such high-performance sensors are rare in IMDs [34-36] and are too energy-consuming for IMDs’ limited battery capacity [37]. Future studies could certainly explore if previous work remains effective at reduced sensor sampling rates such as a few hundred Hz. Nevertheless, this is likely to significantly impact the performance because vibration signal demodulation often requires sensor data with high resolution [29].

Overall, we find that only [31] demands a lower sampling rate of 250 Hz. This is because the secret relies on the resonant frequencies of the user’s hand-arm region, which are situated in the low-frequency domain ranging from several to a few hundred Hz [38,39]. Nonetheless, its practicality was only validated for wristbands but has not been tested in other deployment environments or with different hardware setups.

#### Suitable Protocols for OOB Channel-Based Pairing

Previous work has extensively proposed using an OOB channel for pairing with resource-constrained devices, including IMDs [5,19,26,40,41]. Typically, the ultimate objective of such pairing is to establish a 128-bit cryptographic key between 2 devices for data encryption [7]. However, these works commonly propose to directly exchange the entire key through the OOB channel, which raises several concerns.

First, OOB channels often have much lower data throughput compared to conventional wireless channels. For instance, the data throughput of the aforementioned vibration-based method [31] is only 7.15 bits per second. As a result, a 128-bit key bootstrap would require at least 18 seconds, potentially posing issues of usability and safety in emergencies. Second, OOB channels face threats from advanced side-channel eavesdropping attacks. For example, a vibration channel might be compromised using microphones in proximity due to acoustic leakage, leading to severe consequences.

To mitigate these concerns, prior work has suggested using a password-authenticated key agreement (PAKE) method [19,42,43], such as Diffie-Hellman Encrypted Key Exchange [44]. PAKE is a cryptographic protocol aiming at exchanging a high-entropy cryptographic key between parties who have previously shared a short and low-entropy secret. This approach allows 2 devices to initially exchange a short bitstring, after which they execute a PAKE to further exchange a 128-bit key. The latter step can be fast and thus largely reduce the impact of the low data rate of OOB channels. In addition, PAKE provides forward secrecy and rules out offline brute-force attacks. This is the approach that we adopt in our work, and therefore we consider that vibration is only to be used to exchange an ephemeral and low-entropy secret between the IMD and the external device.

### Objectives

The objective of this paper is to explore the potential of using vibration for pairing with resource-constrained IMDs. This study aimed to (1) propose a novel technique that leverages vibration to extract secrets from the naturally random human motor behavior for pairing, (2) develop a prototype as a proof-of-concept to demonstrate our technique, and (3) evaluate our prototype’s accuracy, security, and usability in a lab study involving 24 participants.

## Methods

### Pairing Technique

The pairing process requires the user (patient or doctor) to repeatedly attach the external device to the patient’s body (near the IMD’s location) for a few times. In this work, each repetition was referred to as a cycle, and the complete pairing process (including several cycles) was defined as a run. Each cycle comprises three main steps:

1. Device attachment: the user attaches the external device to the body and holds it steadily.
2. Vibration broadcast*:* the external device emits a vibration signal for a short period. The signal is always the same and does not serve as the secret. Both the IMD and external device take a measurement of the acceleration. The user releases the external device when the vibration stops.
3. Randomness extraction: both devices process the sensed acceleration signal and derive a shared secret from it.

The security of pairing relies on the randomness of the shared secret, which originates from the diverse physiological characteristics of the human body as well as the inherent variability of human behavior (eg, the varying attachment position and the grip strength) [45]. The vibration signal itself remains constant in each cycle and is not a source of randomness. Instead, it serves as a “catalyst” that allows the randomness of body and motion to be reflected in the accelerometer measurements.

### Obtaining a Shared Secret From Humans

The design of vibration strategy in each cycle—namely, the control of the motor to vibrate at a certain frequency for a certain time frame—is crucial. The feasibility of the aforementioned work [31] was first explored in the context of IMDs. The exact same experimental settings were replicated using our prototype that simulates the human body environment (elaborated in the following sections): the accelerometer sampling rates of the external device and IMD are set as 250 Hz. In each cycle, the motor is programmed to sweep between 20 Hz to 125 Hz within 1.75 seconds. During this period, 2 devices measure the z-axis acceleration data (aligning with the user’s sagittal plane) and subsequently generate the frequency spectrum by doing fast Fourier transform (FFT) [46].

**Figure 2.**
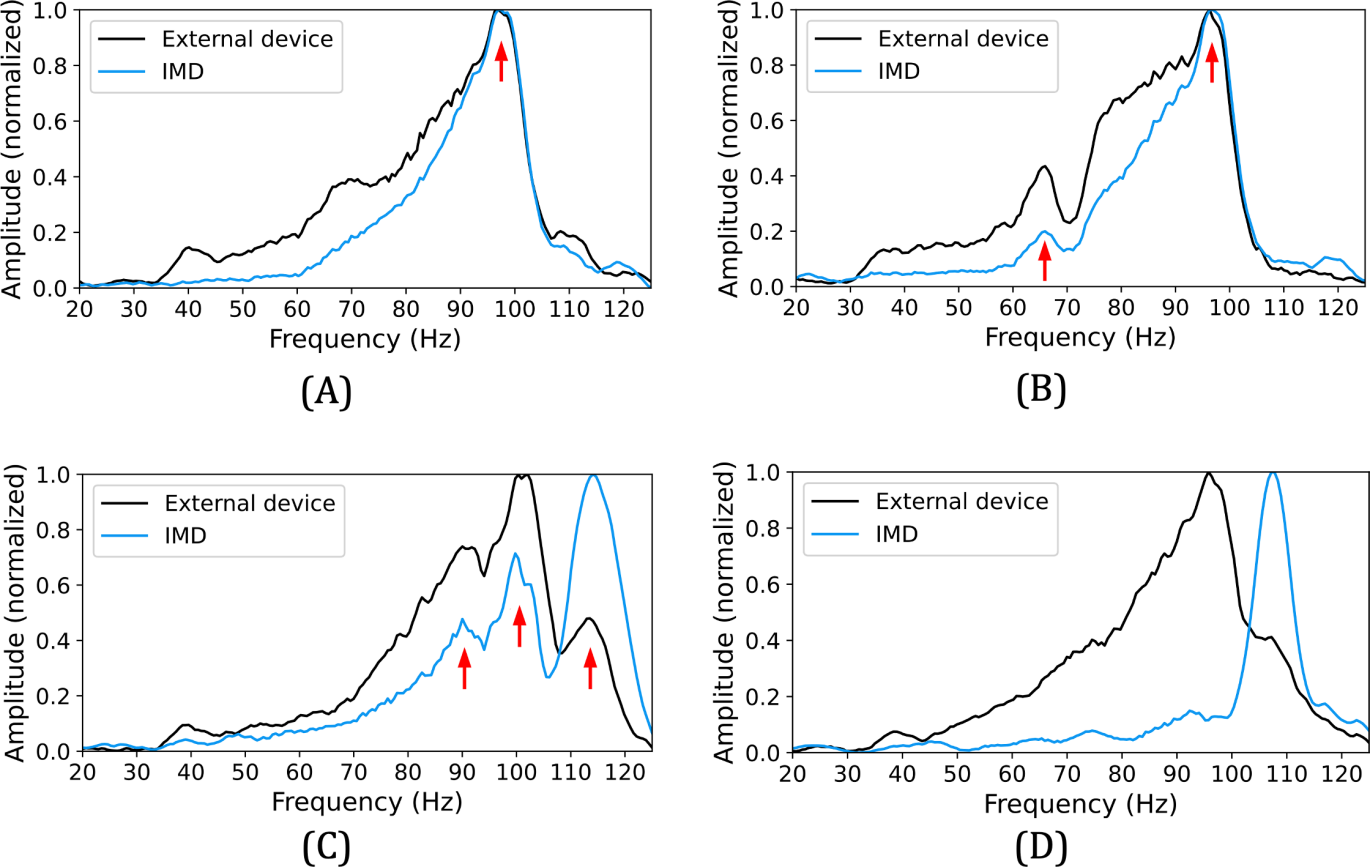
Performance of the preliminary test (A) with 1 peak (72%), (B) with 2 peaks (17%), (C) with 3 peaks (2%), and (D) the noisy data (9%). IMD: implantable medical device.

One researcher of the team performs 100 cycles as a preliminary test. The results are shown in Figure 2 (the locations of the resonant frequency peaks shared by both devices were regarded as secrets in [31]). Among all, 72 cycles show one stable peak; 17 cycles have 2 common peaks; 2 possess 3 peaks; in 9 cycles, the data is too noisy to capture any shared peaks. The results differ significantly from [31] where an average of 4‐8 peaks can be obtained per cycle. In addition, the peaks in [31] are somehow uniformly distributed over the whole 20‐125 Hz range, while ours are almost always in the range of 80‐110 Hz. Our interpretation for the discrepancy in the performance of this strategy is the presence of the plastic board and shell in our prototype setup, which “masks” the resonant frequencies of the human body. Unfortunately, in the context of IMD pairing, the existence of such components (eg, a plastic or metal device housing) is inevitable.

**Figure 3.**
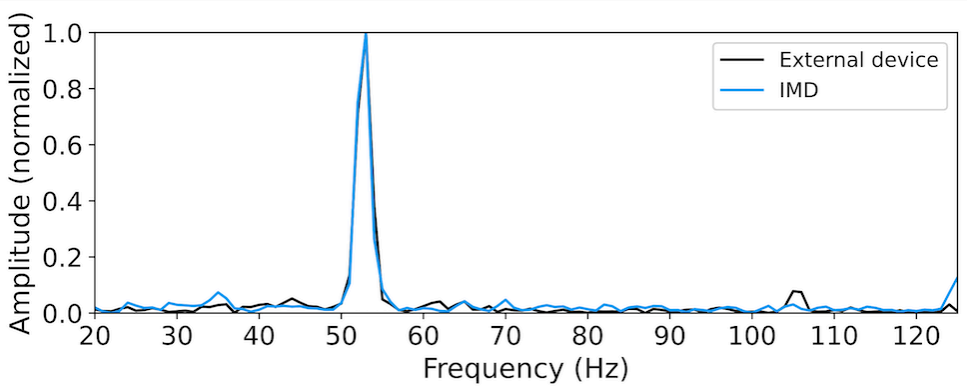
Frequency spectrum given a constant vibration (50 Hz, 1 s) in one cycle. IMD: implantable medical device.

**Figure 4.**
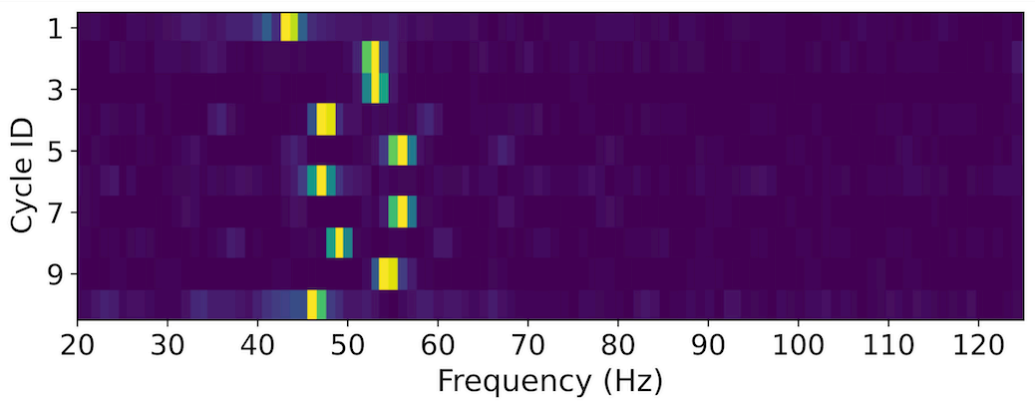
Frequency spectrum of IMD, given a constant vibration (50 Hz, 1 s) in 10 consecutive cycles. IMD: implantable medical device.

Nevertheless, the above test implies the natural randomness inherent in the user attachment motions. Intuitively, we want to test if a constant-frequency vibration is a viable option. We program the motor to emit a 50 Hz vibration for 1 s per cycle, and the same researcher executes 100 cycles using our prototype. For each cycle, we collect z-axis acceleration data from both devices and generate the frequency spectrum using FFT. Figure 3 shows an example of the frequency spectrum in one cycle. It was observed that 2 devices can obtain very similar data, especially for a prominent amplitude peak. Figure 4 illustrates the spectrum change of the IMD over ten consecutive cycles. Each row in this figure corresponds to a frequency spectrum obtained in 1 cycle, and the bright spots indicate the prominent peaks on the curve. We observe that the peak locations vary around 50 Hz, suggesting the presence of a degree of randomness. These findings indicate that providing an excitement of a constant-frequency vibration, the prominent peak location in the frequency domain is a potentially qualified shared entropy source between the IMD and the external device, which can be used for pairing purposes.

### Signal Processing Workflow

**Figure 5.**
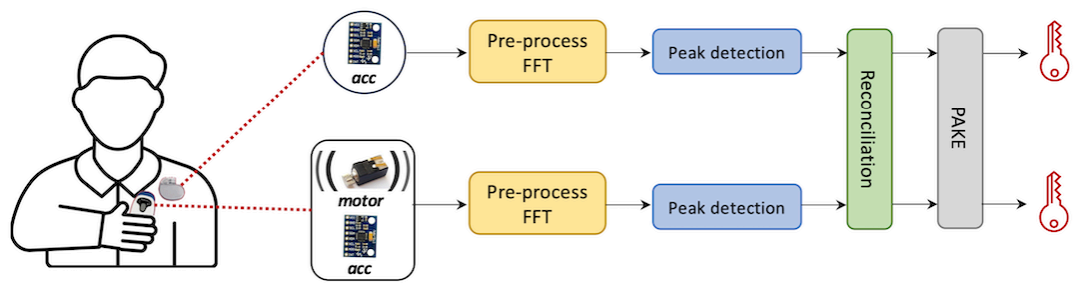
An overview of our pairing technique. acc: accelerometer; FFT: fast Fourier transform; PAKE: password-authenticated key agreement.

Figure 5 shows the workflow of our pairing technique (assuming the IMD is a pacemaker). In each cycle, the patient holds the external device and attaches it on their chest. During the attachment, the motor vibrates, and both the IMD and the external device measure a pair of z-axis acceleration data.

To remove the noise of the direct current component, each device subtracts the acceleration data with its mean value. In addition, when the vibration motor is switched on from standstill or switched off, the generated vibration signal is not amplified or attenuated immediately but with a slow and damped response [26,27]. This means that the transition parts (ie, 2 ends) of a vibration signal segment are often noisy. This is addressed by applying a Hanning window on the data.

Subsequently, each device applies FFT on the acceleration signal to obtain the frequency spectrum. The frequency range of 0 to 20 Hz is then excluded to avoid the effects of noisy motion artifacts like human breathing movements, as well as ambient vibrations present in the patient’s environment [47]. As mentioned, there is a prominent amplitude peak in the frequency spectrum. In order to detect the location of this peak, each device simply traverses the frequency domain to find the frequency value corresponding to the maximum amplitude.

Based on the above procedure, after the user completes a pairing (ie, a run) by repeating the attachment for several times, each of the 2 devices will possess a sequence of peak locations. However, these sequences may not be exactly the same due to the measurement noise and human error (eg, hand wobbles). To resolve this, the peak locations are encoded into binary format using Gray code [48]. This coding method ensures minimal bit mismatches if the discrepant peak locations are very close on 2 devices, which is the case of our technique. Then, we use a cryptographic algorithm known as a fuzzy extractor [11,49] to reconcile any remaining bit differences between the 2 bitstrings without revealing the secret itself. If the rate of bit mismatches falls within the error-correcting capability of the fuzzy extractor, the IMD and the external device agree on an identical bitstring as a shared secret.

### Adversary Model

Given our review of relevant literature about IMD pairing techniques [3,12,13,16,19,40,50], we assume a sophisticated adversary following the Dolev-Yao model [51] who has full knowledge of our pairing protocol, has full control over the wireless communication channels, and can be a man-in-the-middle (MITM) attacker by intercepting legitimate devices’ signals and sending their own messages instead. In particular, the adversary can launch the following attacks relevant in the context of our pairing technique: (1) impersonation attack: the adversary uses a sequence of peak locations in an attempt to impersonate a legitimate device. They could succeed if their sequence closely matches the one measured by the IMD or external device. (2) Brute-Force attack: the adversary brute-forces possible peak location sequences and launches MITM attacks to decipher and manipulate the communication between legitimate devices. The brute force can be done online, that is, during the pairing process, the adversary tries every possible sequence until they hit a correct one. Alternatively, this can be done offline, where the adversary records the pairing traffic and performs offline analysis to crack the secret after pairing. (3) Acoustic eavesdropping: The adversary may also attempt to eavesdrop on the vibration signals using a microphone near the patient to reveal the secret.

### Experimental Setup

The proposed pairing technique was validated through the design and testing of the prototype in a user study. It was assumed that the IMD is a pacemaker implanted beneath the chest and considered the external handheld device to resemble a smartphone with a plastic casing. Moreover, both devices contain an accelerometer, and the external device is equipped with a vibration motor.

### Prototype Implementation

**Figure 6.**
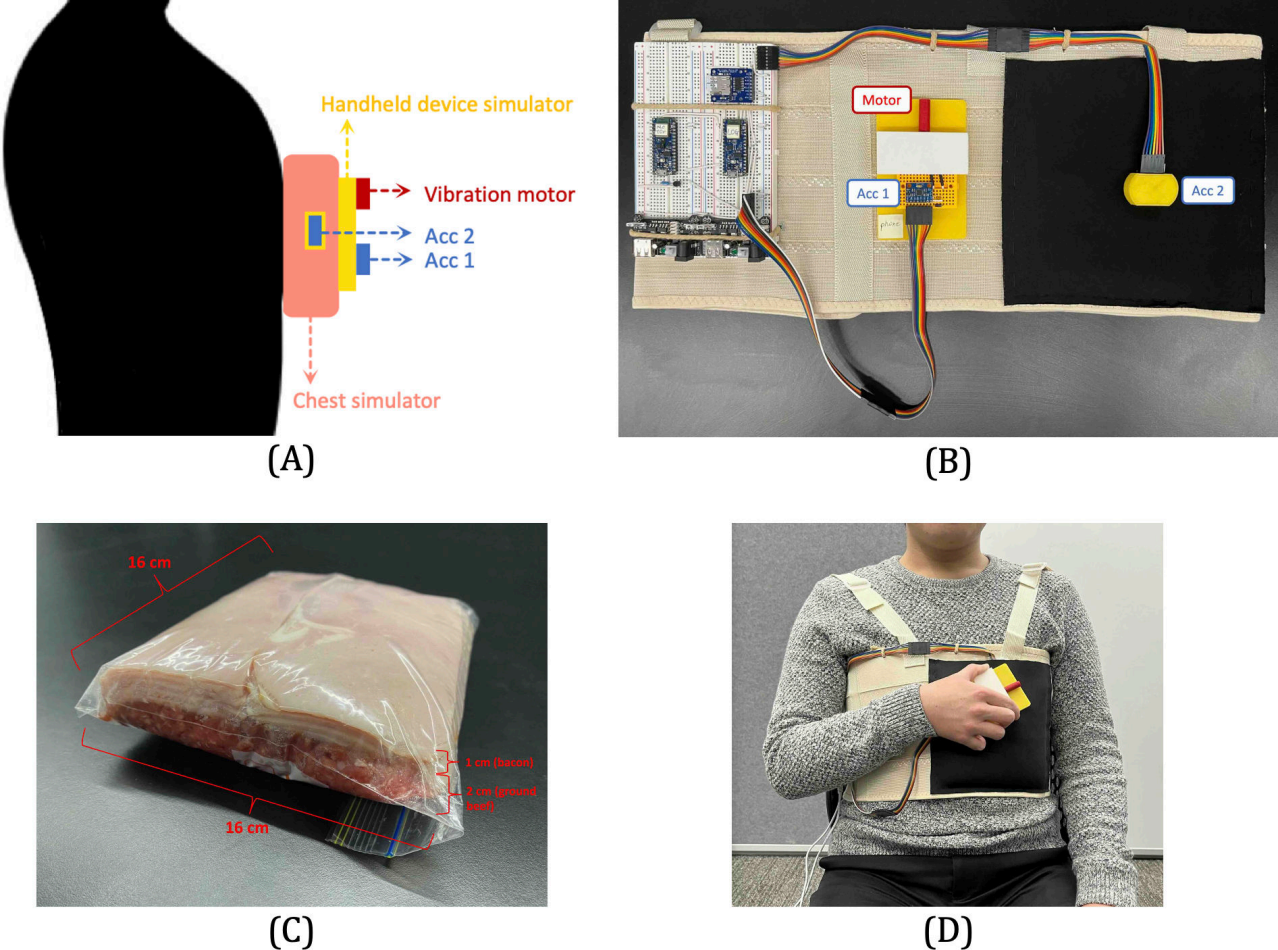
Experiment setup. (A) Prototype overview. (B) Hardware setup. (C) Chest simulator. (D) Participant in user study.

We show an overview of our prototype in Figure 6A. The prototype consists of three main parts:

#### IMD

We use an InvenSense triaxial MPU-6050 accelerometer [52] to simulate a pacemaker and house it inside a 3D-printed case (Acc2 in Figure 6B). An Arduino Nano 33 BLE board interfaces with the sensor, which contains a 32-bit Cortex-M microcontroller and closely resembles the capabilities of an IMD [53]. The sampling rate of the accelerometer is set at 250 Hz, the same as in previous work [31].

#### External Device (the Vibration Transmitter)

We do not directly use a smartphone as the external device because the most common operating systems on mobile devices—Android and iOS systems do not provide an API interface for direct control of the vibration motor frequency. Instead, we use an eccentric rotating mass type vibration motor [54], along with another MPU-6050 sensor (Acc1 in Figure 6B) to simulate an external device. These components are mounted on an 11 cm × 7 cm × 0.5 cm plastic cuboid board, replicating the size and shape of a typical smartphone.

We use a separate Arduino Nano 33 BLE board to control both the vibration motor and the accelerometer. Particularly, this Arduino board connects to the vibration motor and supplies voltage to it. By using the pulse width modulation technique [31], the board can adjust the driving voltage, allowing the vibration motor’s frequency to be altered accordingly. In addition, the accelerometer is set to a sampling rate of 250 Hz.

#### Chest Environment

Given that pacemakers are embedded inside the body, it is important for our experiments to mimic an environment that resembles the human chest. We adopt the design in previous research [5,26,40] and use 1 cm layer of bacon and 2 cm layer of lean ground beef to replicate the chest’s physical properties (see Figure 6C). The 1 cm depth is a standard depth for pacemaker implantation [55]. In our study, we embed our pacemaker simulator within the meat layers, which are kept inside a food storage bag at room temperature. This bag of meat is subsequently placed in a pocket stitched onto an elastic chest band, positioned around an area corresponding to the human heart’s location (see Figure 6B). Participants were asked to wear the chest strap throughout the user study to mimic the conditions of pacemaker users.

### Participant Recruitment

We first conducted a pilot study with 6 individuals (ages 22 to 32 years, 4 females and 2 males) to identify and resolve any problems with our experimental setup. Subsequently, we recruited 24 participants for the main study, including 11 males and 13 females of ages ranging from 18 to 52.

Moreover, given that patients who carry IMDs are often seniors [56], we also conducted a co-design workshop with 2 senior individuals who had intimate knowledge and experience with pacemakers: (1) a 74-year-old female cardiology doctor and (2) a 79-year-old male pacemaker patient.

### Experiment Procedure

In total, 2 essential vibration settings, frequency, and duration, were manipulated to measure the effect on pairing performance. Based on experiences gained from our pilot study, we set vibration motor frequencies to 50 Hz, 75 Hz, and 100 Hz, and vibration durations to 400 ms, 700 ms, and 1000 ms. The 9 frequency–duration combinations enabled successful pairing and avoided excessive participant workload.

During the user study, participants were instructed to wear our prototype and sit on a chair. Then they need to grasp the external device simulator and repeatedly attach it to the black pocket area of the chest strap, as shown in Figure 6D. They were advised to attach the device in a random manner (such as to random positions), and (in each cycle) stay attached until the vibration had completely ceased. Before starting the data collection, participants were asked to acquaint themselves with the prototype to understand the pairing process. This introductory process took under a minute for all participants. Subsequently, for each of the 9 vibration conditions, participants were asked to conduct the attachment for 5 consecutive cycles as one run and complete 4 such runs in total. The order in which participants used different vibration frequencies was counterbalanced.

At the end of the user study, participants were requested to fill out a standard system usability scale (SUS) questionnaire [57] to assess the usability of the pairing method. We then conducted an interview with them to gather further insights. Full details of the questionnaire and interview are given in Multimedia Appendix 1.

During the co-design workshop, we asked the two senior participants to try our prototype for only 6 runs (considering their physical conditions) and provide their opinions and advice.

### Evaluation Metrics

Our study focuses on certain metrics to evaluate the pairing performance.

#### Accuracy

The accuracy of a pairing system is typically measured by false rejection rate (FRR) and false acceptance rate (FAR) [43,58,59]. FRR is the frequency at which the pairing between legitimate devices is incorrectly rejected. FAR indicates the frequency that a pair of illegitimate devices (such as the IMD and a malicious external device) is mistakenly authorized and gauges the resilience of pairing against impersonation attacks. A high FRR and FAR could lead to poor usability and security, respectively. These 2 metrics are calculated as follows:


$$FRR=\frac{Number\;of\;rejected\;pairing\;between\;legitmate\;devices}{All\;pairing\;attempts\;between\;legitmate\;devices}$$

$$FAR=\frac{Number\;of\;authorized\;pairing\;between\;illegitmate\;devices}{All\;pairing\;attempts\;between\;illegitmate\;devices}$$

During the pairing process, there is often a mismatch (denoted by *d*) between the readings of the IMD and the external device due to inherent noises. As aforementioned, we use a fuzzy extractor scheme to correct the mismatch. At the core of this method is the selection of a threshold (denoted by Thr): the mismatch can be rectified (and thus the pairing is accepted) if *d*≤Thr; otherwise, the pairing is rejected. As such, one can balance FRR and FAR by adjusting Thr. Because security is of utmost importance for the IMDs, we set a smaller Thr to ensure FAR=0 and use the corresponding lowest FRR to represent accuracy.

#### Security

The FAR metric evaluates the system’s security against impersonation attacks. The resilience against brute-force attacks is determined by the randomness level of the attachment motions, which can be measured in two primary ways: (1) By the National Institute of Standards and Technology (NIST) statistical test suite [60] that provides a comprehensive randomness assessment of a random number generator, a method widely recognized within the cybersecurity community [19,31,43]. (2) By measuring Shannon entropy, which quantifies the amount of information contained in each motion event [17,31,61,62].

#### Usability

Usability is assessed based on the results from our SUS questionnaires and interviews.

### Ethical Considerations

This study involved human participants and underwent thorough ethical review, particularly given the potential involvement of older participants. Ethics approval was obtained from the relevant institutions prior to participant recruitment and user study, in accordance with institutional regulations (the University of Melbourne: approved by the Human Ethics Committee, application number: 2022-24851-31088-3; the University of Birmingham: approved by the Science, Technology, Engineering and Mathematics Ethics Committee, application number: ERN_2022‐0255).

Participants were recruited via online advertisements and were offered US $30 for their time. All participants provided informed consent prior to participation. The data collected during the user study were specifically processed to ensure anonymity and untraceability of identity and were securely stored in the University of Melbourne’s data center. All participant data were anonymized by removing personally identifiable information before analysis, and participants were assigned unique identification codes to ensure confidentiality. The entire user study process was overseen by a departmental delegate of the university’s ethics committee, with all study details reported to them on a weekly basis.

## Results

### Performance of the Pairing Technique

**Figure 7.**
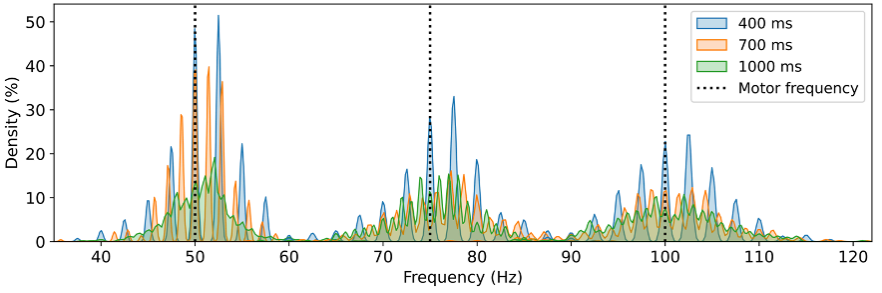
Distribution of peak location measured by the IMD among 24 participants. The black dashed lines indicate frequencies of the vibration motor. IMD: implantable medical device.

Figure 7 shows the distribution of all peak locations (ie, the secret) collected by the IMD from 24 participants. We observe that for a specific vibration frequency, such as 50 Hz, the peak locations range between 30 and 70 Hz and generally approximate a normal distribution centered by the motor’s frequency, suggesting a certain degree of randomness from the user. Additionally, it appears that the distribution is slightly flatter (thereby the level of randomness increases) with an increase in vibration frequency. Notably, the possible options for peak locations in the frequency domain are not continuous due to the sample-based nature of the time domain acceleration data.

**Figure 8.**
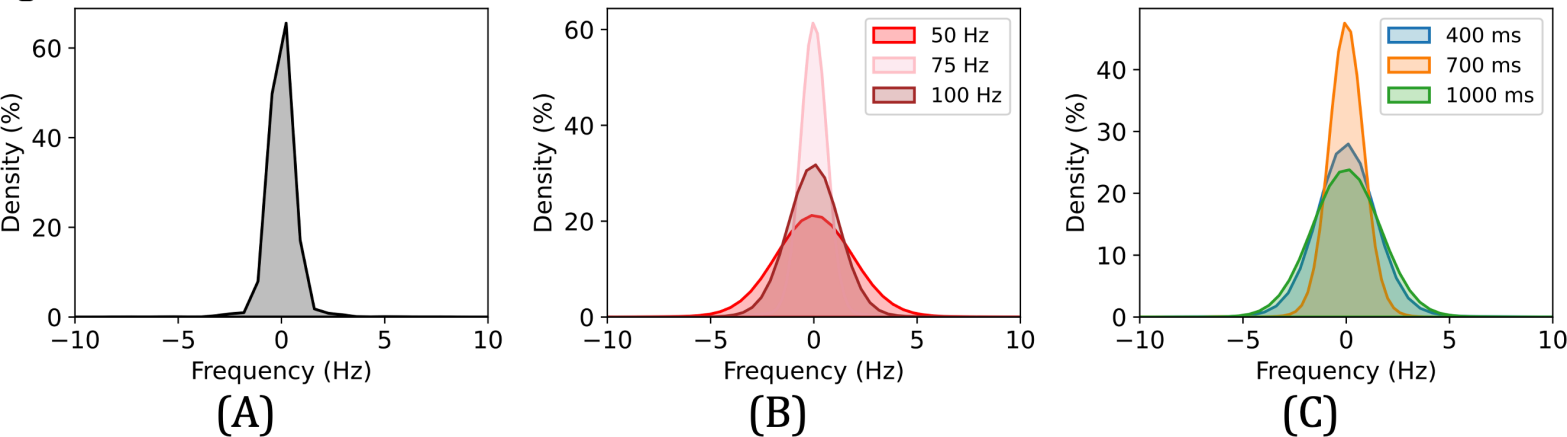
Mismatch between the IMD and the external device. (A) Mismatch of all data among participants. (B) Mismatch with vibration frequency. (C) Mismatch with vibration time. IMD: implantable medical device.

Mismatch is calculated by subtracting peak location values between the IMD and the external device and represents the level of noise and error. The mismatch distribution for our prototype, as illustrated in Figure 8A, resembles a normal distribution centered around a mean near zero and with a standard deviation of 2.8 Hz. This implies that user-induced errors and sensor noise are limited. Note that this result considers situations where participants did not strictly follow our pairing norms during the study. For instance, there were a number of occasions when participants released the external device while it was still vibrating. Such cases were not excluded from our dataset as they present a more realistic use scenario; otherwise, we expect that the mismatch levels would be even lower. On the other hand, Figure 8B and C show that the degree of mismatch does not have a straightforward correlation with either the vibration frequency or the duration.

### Experimental Evaluation

#### Accuracy Assessment

For each of the 9 vibration conditions, we build two sets to measure accuracy:

Set I comprises 96 (=24 × 4) pairs of peak locations, each with a length of 5 (since we collected 5 cycles per run). All the pairs in Set I come from legitimate pairings of an IMD and an external device. This set calculates the FRR metric as aforementioned.

Set II has 96 pairs of peak locations (the same size as Set I), where each pair is created by randomly mixing data from illegitimate device pairings. This set calculates the FAR metric as aforementioned.

An effective pairing technique should maximize the acceptance of pairs from Set I (ie, low FRR), while minimizing the acceptance of pairs from Set II (ie, low FAR). Note that not all five motions are necessarily needed, ie, we can vary the length of runs ranging from 1 to 5, by truncating the initial elements.

The following figures show the accuracy of our prototype across various numbers of attachment motions performed. FAR is 0 in all cases, and we consider that an FRR below 5% signifies good usability [43,61]. As expected, increasing the number of motions consistently improves the accuracy of the pairing. Moreover, given a specific vibration frequency, longer vibration duration leads to higher accuracy, which will be further discussed in the coming sections. An additional observation is that with a fixed vibration duration and number of motions, the FRR tends to drop as the vibration frequency rises.

**Figure 9.**
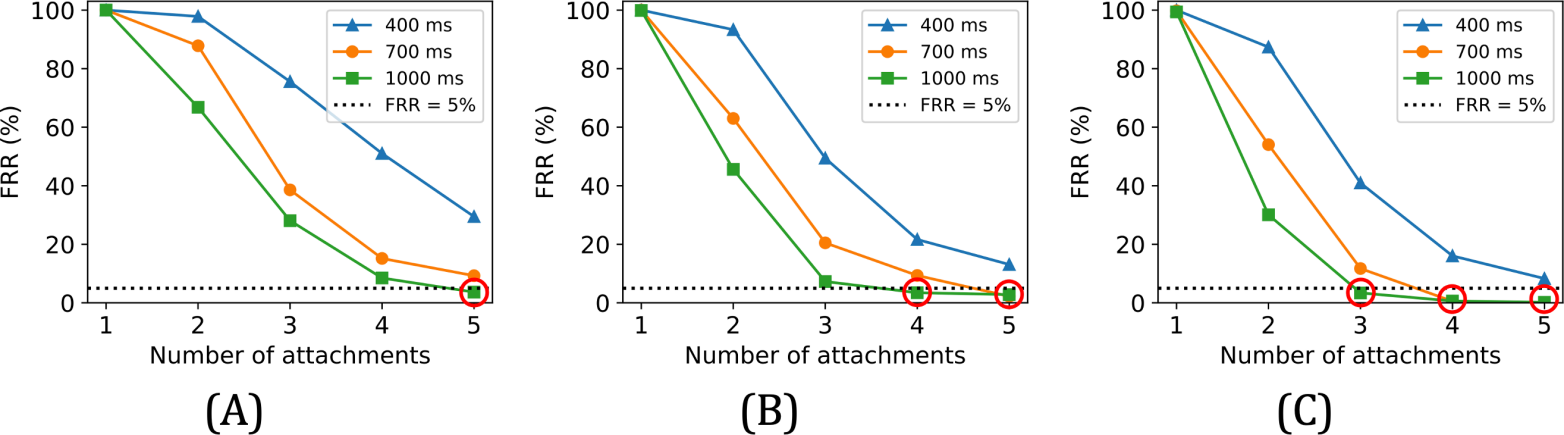
FRR versus number of attachments under different vibration frequencies: (A) 50 Hz, (B) 75 Hz, and (C) 100 Hz. FRR: false rejection rate.

Overall, the red circles in the above figures indicate the 5 out of 9 vibration conditions that offer acceptable accuracy levels (with FAR=0 and FRR <5%). For example, a vibration condition of 50 Hz for 1000 ms per cycle requires the user to execute five attachments to achieve pairing with FAR=0 and FRR=3.7% (see Figure 9A). Note that for other vibration conditions, more than five motions are likely to also yield satisfactory accuracy. However, this would demand more effort from the user, which could harm usability and even safety in emergencies.

#### Security Assessment

We refer to previous work [19,31,43] to assess the randomness of the secret generated by our technique: For each of the vibration conditions, we take the floor of the (fractional) entropy value for that specific setting (refer to Table 2) and extract that number of least significant bits from each peak location value. Subsequently, we combine these bitstrings from all vibration conditions (following the order in our user study) as a single 8.6 kbits string and evaluate its randomness using the NIST statistical test suite [60]. The full results are given in Table 3. The outputs of the NIST tests are *P *values that represent the probability the data is generated by an eligible random number generator. If a *P* value is smaller than a threshold (usually .01 [19,31,43]), the randomness hypothesis is rejected. Table 3 shows that all *P* values are larger than .01 and hence pass the NIST tests.

**Table 2.** Entropy of each attachment motion (unit is bit).

	50 Hz	75 Hz	100 Hz
400 ms	2.61	3.12	3.87
700 ms	3.03	3.86	4.15
1000 ms	3.15	4.01	4.48

**Table 3.** NIST statistical test results for attachment motions.

Test	*P* value	Test	*P* value
Frequency	.88	Block frequency	.14
Runs	.11	Longest runs	.10
Binary matrix rank	.17	FFT^a^	.62
Non-overlap template	.16	Overlapping template	.38
Serial (*P* value_1_)	.64	Linear complexity	.72
Serial (*P* value_2_)	.68	Approximate entropy	.18
Cumulative sums (forward)	.19	Random excursions	.22
Cumulative sums (reverse)	.26	Random excursions var.	.40

a FFT: fast Fourier transform.

Table 2 shows the entropy value contained in each motion across different vibration conditions. Overall, a single motion in our study carries an entropy from 2.61 to 4.48 bits. For a certain vibration frequency, the entropy grows with higher vibration durations. This is because extended measurements yield larger sample size and frequency resolution, enabling more possible peak locations and thus higher entropy. Furthermore, for a given vibration duration, the entropy value rises with an increase in vibration frequency. We leave the study of this phenomenon to future work. Nevertheless, the choice of vibration frequency is often limited by the capability of the motor and accelerometer in practice.

It is noteworthy that some entropy is sacrificed when rectifying mismatches between the 2 devices. Here, we make a preliminary estimation of the entropy loss: Using the encoding method in [43] on our dataset, the maximum bit mismatch rates (ie, percentage of different bits between two devices) for our prototype vary between 0.7% and 3.0% for different vibration conditions. This can be addressed by a fuzzy extractor with (31, 29) Reed-Solomon code that has a 3.23% error tolerance [11,63], potentially leading to an entropy loss of 6.5%.

#### Usability Assessment

The average SUS score for our pairing technique is 73.6 (SD 18.14), which generally passes the typical benchmark value of 68 for “good usability” [57]. It is important to note that the SUS questionnaires were completed after an extensive data collection process including a repetition of 180 attachment motions. We expect that users carrying out a more realistic task would report even higher usability scores.

We gained further insights into usability from the interviews. Over half of the participants (15 out of 24) explicitly indicated that our technique was easy to use. For example, one participant (p8) commented, “The attachment doesn’t require me to think. This is an advantage. I don’t know what is happening here, but I prefer it as it requires less effort,” and another participant (p13) remarked, “It’s easy. You don’t really have to move that much, and you can do it while you’re sitting as well.” Some participants expressed their preference for the vibrational feedback. One participant (p1) said, “The vibration is good feedback, and I don’t have to visually see anything,” and another participant (p22) noted, “The process is like listen to my heart.” In addition, some participants conveyed that they found the pairing process to be enjoyable and fun. For example, 3 participants described the vibration as a hand massage and 2 compared the pairing activity to using a stethoscope.

Most participants (18 out of 24) experienced no discomfort during the study. Nonetheless, the rest of the 6 people did report some discomfort at the end of the study. In total, 4 participants noted that the intensity of the vibrations was excessive; for example, one participant (p4) stated, “I feel like my entire chest is vibrating, and I don’t like the feeling.” This concern might be resolved by selecting a vibration motor with lower amplitude. In addition, 4 participants reported feeling fatigued after the data collection process, but also noted this was due to the repetition of 180 attachments and that less motions will alleviate this issue. Furthermore, one participant (p3) criticized the prototype design and mentioned that the external device simulator was too big. We leave the refinement of our prototype as future work.

Valuable insights were also gathered from the co-design workshop. Both participants initially found the vibration-based pairing technique interesting and somewhat surprising, but they quickly became accustomed to it and could easily complete the remaining required motions. They both explicitly noted that the pairing operations were easy to learn and perform. The cardiology doctor described the pairing operation as “using a stethoscope” and confirmed that the vibration signal in the experiment would pose minimal risks to patients with IMDs. Both participants also appreciated the tactile feedback from the vibrations. The doctor commented, “The vibration tells you if you’re on track,” while the pacemaker patient added, “The vibration encourages me towards the end of pairing.” However, both participants pointed out that the prototype used in the study was bulky and heavy—an improvement we leave for future work. Overall, both participants found the pairing experience acceptable and expressed willingness to use it in real-world scenarios if required.

#### Optimal Setups

Based on our analysis so far, we summarize all pairing configurations that (1) exhibit high accuracy with zero FAR and FRR under 5%, and (2) generate a level of entropy surpassing a standard four-digit PIN code (with an entropy of 13.3 bits), which is commonly used in pairing of Bluetooth technologies and other security systems [8]. All viable settings that meet these requirements (with minimum required number of motions) are shown in Table 4. Note that the time values include both the vibration duration and an additional “preparation time” necessary for a user to detach and then reattach the external device to their body; in our study, this interval was 0.5 seconds.

In summary, we find that with a vibration configuration set at 100 Hz and 700 ms, a user can carry out 4 attachment motions to enable the exchange of a secret with (FAR, FRR)=(0, 0.6%) and entropy of 15.5 bits. This process can be completed in a mere 4.8 seconds.

**Table 4.** Summary of well-performing pairing configurations.

Vibration condition	Motion, n	FAR^a^, FRR^b^ (%)	Entropy	Time (s)
50 Hz, 1000 ms	5	0, 3.7	14.7	7.5
75 Hz, 700 ms	5	0, 2.2	18.0	6
75 Hz, 1000 ms	4	0, 3.5	15.0	6
100 Hz, 700 ms	4	0, 0.6	15.5	4.8
100 Hz, 1000 ms	4	0, 0.6	16.8	6

a FAR: false acceptance rate.

b FRR: false rejection rate.

## Discussion

### Principal Findings

Our work introduces a new and reliable vibration-based pairing approach for IMDs, which only requires a low sampling rate accelerometer and relies on the natural randomness inherent in human behavior. We empirically validate the feasibility of our technique through a user study. Overall, we find that the workload required to bootstrap a secure pairing is minimal, and we estimate that it requires the user to attach a device to the body only 4 times in roughly 5 s. With an FAR of 0 and an FRR of 0.6%, the risk posed by adversaries is low, and legitimate users will likely experience very few failures.

As mentioned in the related work section, the use of a PAKE eliminates offline brute-force attacks. In addition, it also restricts the number of online MITM attempts. Typically, the adversary has a very limited period to obtain the secret and usually only one chance for a MITM attack [44]. As an estimate, 4 motions with 15.5 bits entropy reduce the adversary’s success probability on online brute-force attacks to 0.002% [42] (assuming the adversary is limited to guessing only). Therefore, we believe these motions serve as adequate input for a PAKE. If needed, higher entropy can be easily achieved by performing more motions.

Our user study confirmed the high usability of our pairing method. Participants found it straightforward to understand, learn, and perform. The process of attaching the device is very intuitive, like using a stethoscope as described by the participants. Our technique also brings about certain entertainment to users, being both relaxing and enjoyable (such as described as hand massage). This could be advantageous in certain therapeutic treatments, where physical interaction can enhance memory, concentration, and mental health [64]. Moreover, it is worth noting that for patients who are unable (eg, due to disabilities or unconsciousness in emergencies) or unwilling to execute the motions, our pairing allows medical practitioners or caregivers (who have received appropriate training) to execute the motions on the patient’s body on their behalf.

Our proposed method only requires an accelerometer, a component already present in the latest generation IMDs [23-25]. The signal processing and other cryptographic algorithms for the IMD are computationally lightweight and work efficiently on 32-bit Cortex-M microcontrollers, which closely resemble IMDs’ capabilities [11,43,53]. Our approach solely depends on vibration at a constant frequency, which can be easily implemented on readily available consumer devices such as smartphones and tablets. This is beneficial considering that medical device companies already equip the IMDs with the ability to connect to personal mobile devices [65]. Moreover, while our work assumes that the IMD is a pacemaker, we argue that the technique can be easily transferred to other types of IMDs or even external wearables. Furthermore, our proposed pairing technique incurs minimal costs. In our prototype implementation, the combined cost of the vibration motor and accelerometer was under $30, and this cost could be further reduced during mass production.

### Comparison With Prior Vibration-Based Work

Our pairing technique significantly relaxes the demands on the IMD’s sampling capability. We use an accelerometer operating at 250 Hz, in contrast to previous work that often relies on sampling rates of several thousand Hz or more. In particular, the sampling rate can be further decreased by using lower vibration frequencies. For example, with a 50 Hz vibration, the frequency domain peaks cluster between 30 and 70 Hz (see Figure 7), indicating that an accelerometer with a 140 Hz maximum is adequate.

Conventional approaches typically try to avoid user-generated noise. For instance, the user needs to ensure stable contact between devices during data transmission. Conversely, our method harnesses user noise and benefits from it as a source of entropy. Indeed, our dataset includes many instances with significant user error, like when a participant releases the external device before the vibration completely stops. In such scenarios, the IMD only captures a portion of the vibration within its measurement window. Despite this, our technique maintains high reliability.

Furthermore, previous work that encodes secrets into vibrations often demands precise time synchronization in milliseconds between devices, which itself is a challenging task for resource-constrained devices [66]. In contrast, our approach allows for more lenient synchronization—as long as the two devices capture similar vibration signals within most of their measurement windows, the peak locations effectively match. This aspect greatly enhances the feasibility of our technique for IMDs.

Notably, our data throughput is significantly lower than [26,27,29,32] and is comparable with [31,33]. Considering the scenario of transmitting a 4-digit PIN code for use in a PAKE, previous work [26,27,29,32] only needs 0.0004 to 0.665 s, which is much faster than the 4.8 seconds required by our method. However, this rapid transmission, while advantageous in many daily applications, may not be suitable for IMD pairing contexts, where the vibration serves not only for secret exchange but also as a crucial cue for patients to be aware of the pairing process. In contrast, we argue that a duration of 4.8 s strikes a balance: it is long enough to be noticeable, yet short enough to maintain usability and safety in emergencies.

### Considerations of Health Implications With Vibrations

Our proposed pairing technique incorporates vibration, a feature that naturally raises concerns regarding the potential long-term health impacts on patients. However, current research indicates that only long-term and excessive exposure to vibrations is linked to adverse effects on mental and physical health [67,68]. In contrast, our method involves brief vibrational interactions, which last less than 5 seconds and may not occur every day. This limited exposure could reduce the likelihood of the negative health consequences.

### Resilience to Acoustic Eavesdropping Attacks

Vibration is essentially a low-frequency audio signal, which inevitably emits acoustic side-channel information that might be eavesdropped using a microphone. This is particularly threatening for methods that encode secrets within vibration signals. For example, Halevi and Saxena [69] found that secrets transmitted this way could be severely compromised using an off-the-shelf microphone from a few meters away. To mitigate this, Kim et al [26] and Anand and Saxena [70,71] proposed using Gaussian white noise or masking signals to obscure the acoustic leaks. These approaches have shown promise in reducing side-channel vulnerabilities against advanced eavesdropping attacks.

In comparison, as shown in [31], the risks associated with eavesdropping are significantly reduced when the vibration is not the carrier of the secret. Our research aligns with this guideline, using a constant vibration signal across sessions to minimize acoustic leakage. In addition, existing countermeasures [26,70,71] are also applicable to our method.

### Limitations

Our work has certain limitations. Our experiments did not explicitly recruit participants who were IMD patients (mainly due to ethics constraints of the institutions where the user study was conducted). Further validation of our approach with these patient groups is necessary.

We designed our prototype in line with previous work in the IMD security community [5,26,40]. However, there is room for enhancement, particularly in its size and weight. Future research should develop more skin-conformable and miniaturized prototypes.

Another aspect of future work is to empirically evaluate the susceptibility of our pairing technique against microphone-based eavesdropping attacks at a distance.

### Conclusion

In this paper, we explore the potential of leveraging vibration to pair with an IMD. We propose a novel technique that uses a straightforward constant-frequency vibration to extract secrets from natural and random human motor behavior for device pairing. We implement and validate our technique through a user study. Overall, we show that it is feasible to establish a cryptographic key in 5 s with high usability, based only on standard vibration motors and accelerometers with low sampling capabilities. The ubiquity of accelerometers in today’s commercial smart devices and IMDs maximizes the chance of acceptance of our design. In general, we hope that our work will serve as a reference for pairing with resource-constrained devices using vibrations in body area networks.

## Supplementary material

10.2196/57091 Multimedia Appendix 1 Questionnaire and interview design.

## Abbreviations

FAR: false acceptance rate
FFT: fast Fourier transform
FRR: false rejection rate
IMD: implantable medical device
IoT: Internet of things
MITM: man in the middle
NIST: National Institute of Standards and Technology
OOB: out-of-band
PAKE: password-authenticated key agreement
SUS: system usability scale
