# Supplementary material for: Using Vibration for Secure Pairing With Implantable Medical Devices: Development and Usability Study
Source: JMIR Biomed Eng. 2025 Aug 26;10:e57091. doi: 10.2196/57091 (PMC12379749; doi:10.2196/57091)
Supplement: Multimedia Appendix 1 [file biomedeng-v10-e57091-s001.docx]

## **Multimedia Appendix 1: Questionnaire and Interview Design**

We use a standard SUS questionnaire, which is widely accepted by the research community to assess usability. It consists of ten questions and provides participants with a five-point scale, ranging from “strongly disagree” to “strongly agree”. The results of the SUS questionnaire can be quantified into a score between zero and 100; a score higher than a threshold (usually 68) suggests good system usability. The complete list of questions is listed below.

(1) I think that I would like to use this pairing method frequently.

(2) I found the pairing unnecessarily complex.

(3) I thought the pairing method was easy to use.

(4) I think that I would need the support of a technical person to be able to pair.

(5) I found the various functions in this pairing method were well integrated.

(6) I thought there was too much inconsistency in this pairing method.

(7) I would imagine that most people would learn how to pair very quickly.

(8) I found the pairing method very cumbersome to use.

(9) I felt very confident using the pairing method.

(10) I needed to learn a lot of things before I could get going with this pairing method.

At the end of the user study, we asked each participant two interview questions to gain further insights. The conversation was recorded and analyzed by the principal researcher. The details of the questions are given below:

(1) Can you share your experiences of using the pairing method?

(2) Have you noticed anything uncomfortable in the pairing process?
